# Institutional Needs

**Published:** 1918-07-27

**Authors:** 


### Institutional Needs.

### SATINETTE BED-SHEETING.

We are very agreeably impressed with the quality of a sample of satinette bed-sheeting sent us by Messrs. J. G. Ingram, the London India-rubber Works, Hackney Wick. All their bed-sheets are fitted with eyelets to prevent them from slipping after being fastened, and by this means the linen over which the sheet is spread is protected. They are made in three drab qualities, satinette, migniard, and listral; the first named is, however, in our judgment, well worth the extra money it costs, since it is guaranteed to resist the action of urine, ammonia, carbolic acid, blood, and chloroform, and is sterilisable by boiling water without deterioration to rubber or fabric.

